# COVID-19 vaccine as a common good

**DOI:** 10.7189/jogh.11.03109

**Published:** 2021-09-18

**Authors:** Gustavo Silveira Borges, Benício Fagner Dos Santos

**Affiliations:** PPGD/UNESC, Universidade do Extremo Sul Catarinense – UNESC (University of Extreme Catarinense South), Criciúma, SC-Brazil

The pandemic has raised concerns from important voices throughout the international scientific community because of the impacts generated by the collapse of health systems in the global scenario of disease emergency caused by the advent of COVID-19. Mohamad Yunus, Nobel Peace Prize winner, for example, idealized the movement *Declare COVID-19 Vaccines a Global Common Good,* which brought together 156 personalities, including 25 Nobel Prize winners, 39 chiefs of state or government, and 86 leaders from the business, cultural, and artistic worlds. It is in this perspective that emerges the movement Declare COVID-19 Vaccines a Global Common Good, and is supported by many voices from different sectors of the global society. The signatories of this movement advocate that vaccines for prevention and immunization against COVID-19 and its variants should be recognized as a global common good, with equal access, regardless of religious beliefs and socioeconomic status. Other In initiatives also support this same idea. Examples of these other initiatives are Covax, within the WHO framework; the Global Alliance for Vaccines (Gavi); and the Coalition for Epidemic Preparedness Innovations (CEPI). In addition to the COVID-19 Tools Access Accelerator (ACT) [1,2].

A global union gained impetus, and made possible the realization of the *Access to COVID-19 Tools (ACT) Accelerator*, which functions as a collaborative structure that raises funds and other resources which enables investment, production, and access to testing, treatment, and immunization against COVID-19. This collaborative model has received support from a variety of sectors. “Governments, health organizations, scientists, companies, civil society, and philanthropists” have contributed financially to allow the production of tests, treatments, and vaccines against COVID-19 to be made available worldwide [2].

In this context, considering the increase of social inequalities and the need to seek solutions that contradict the neoliberal model of treating health as a product, the connections between the human right to health and common goods are recognized as an alternative to the outdated dichotomy of public vs private. Thus, health goods and services from the point of view of common goods work as a way to realize the principles of protecting human rights, and, at the same time, serve as a project of political, economic, and social emancipation [3].

The collapse of health systems, or its prediction, has demanded significant efforts from government authorities and other entities around the world to seek alternatives that prevent its collapse in situations such as the emergence of a global pandemic. In this sense, the necessity to prevent the overload of the systems is the highlight of the moment. Moreover, this gives amplitude to the discussions about health as a human right. Furthermore, being aware of the repercussions that an epidemiological pandemic brings along, several people within the global community point to the urgent and necessary development of a vaccine capable of stopping the damaging effects caused by the disease. In this context, the connection between solidarity and human rights reveals itself to be of singular importance. The evident interconnection between populations, especially in situations of vulnerability, that occurred and continue to occur with the pandemic, has served to expose not only the fragility of health systems, but also, economic models that ignore the very human essence of producing and managing, in a shared manner, the goods and resources necessary for the maintenance of the species itself [4,5].

**Figure:**
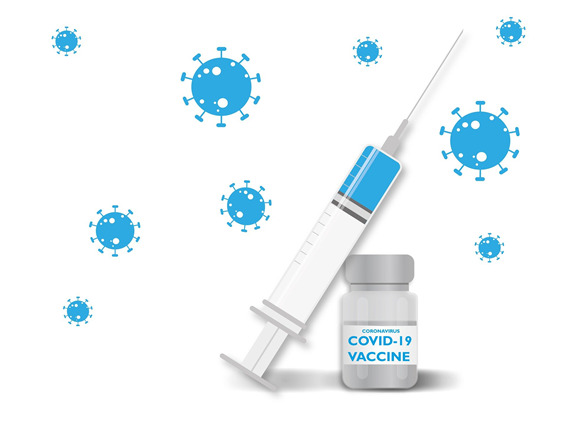
Photo: Alexandra Koch, via https://pixabay.com/pt/illustrations/seringa-vacina-inje%c3%a7%c3%a3o-vacina%c3%a7%c3%a3o-5882593/.

Many voices have advocated that the effects of the COVID-19 global pandemic can only be overcome through immunization on a global scale. They argue that the equitable global production and distribution of vaccines are necessary and that peoples should have access to the different types of vaccines developed so far, as well as to those yet to emerge. Some global movements that evoke the vaccine as an essential resource to human existence itself can be identified from collective practices whose interests are intrinsically focused on the production and protection of those goods and resources that they perceive as essential. The vaccine is placed in the category of common goods based on the idea that the “common can be anything that a community recognizes as capable of satisfying a truly fundamental need not met by market exchanges” [6-8].

Moreover, from this perspective, we see that there is a clear connection between solidarity and human rights, which becomes more evident in the pandemic scenario, where the interconnection between people has become clear in the face of situations of global vulnerability. From this perspective, we see there is a clear connection between solidarity and human rights, which becomes more evident in the pandemic scenario, in which people's interconnection becomes clear in the face of situations of global vulnerability. Thus, it allows the rediscovery of the principle of solidarity that leads to new practices of the commons [9].

These observations lead us to infer that the simple approval of vaccines to control the evolution of the COVID-19 virus is not enough to solve the abyss caused by its social variant, which is inequality. In this way, it is essential to overcome four dimensions or perspectives about the vaccine: first, we must work for its release for using in humans as soon as possible, following the safety protocols recommended worldwide; second, affordable prices, enabling the achievement of the third and fourth dimensions, which are the allocation and availability of vaccines, respectively. In addition, from this understanding, it is possible to reach the idea of the COVID-19 vaccine as a common good. Regardless of the origin of the resources that finance the development and production stages of the vaccine, this resource should be moved to a category of common goods, enabling its protection from exclusionary market models. Furthermore, it, necessarily, implies recognizing access to the vaccine as a fundamental human right, under the risk of violation of this right, but also of human existence itself [10].
